# Feeling Disconnected: River Fragmentation Alters Parenting, Aggression, and Risk‐Taking in Threespine Stickleback

**DOI:** 10.1002/ece3.72099

**Published:** 2025-09-15

**Authors:** F. Leri, L. R. Stein

**Affiliations:** ^1^ School of Biological Sciences University of Oklahoma Norman Oklahoma USA

**Keywords:** aggression, habitat fragmentation, parental care, risk‐taking, threespine stickleback

## Abstract

Habitat fragmentation is a global challenge stemming from human‐induced change. As habitat fragmentation is expected to worsen over time, there is a need to identify organism traits that can predict population persistence within fragments. Behavioral traits are primary candidates for understanding population response to fragmentation because behavior often serves as a first response to changing conditions. While behavior is a topic of interest in the context of habitat fragmentation, many studies are limited to investigating movement patterns. Parental care behaviors are directly tied to individual fitness and offspring survival, which may provide predictive value to population persistence under fragmentation. However, it remains unclear both whether and how parental care differs in organisms found in fragmented areas. We utilized the threespine stickleback ( *Gasterosteus aculeatus* ), a fish with obligatory paternal care, to examine the effects of aquatic fragmentation on parental care, territorial aggression, and juvenile risk‐taking behavior. Following the identification of fragmented (pooled) and non‐fragmented (connected) sites, we recorded parenting behaviors necessary for offspring survival. We then exposed territorial and parenting males to both a known and unknown conspecific intruder to assess aggression. Finally, juveniles were measured in a risk‐taking assay. Adult stickleback in fragmented sites showed a reduction in parental care and heightened aggression toward unknown conspecific intruders, while juveniles in fragmented sites were more hesitant to emerge into a novel environment. Altogether, our results provide support for changes in parental care within habitat fragments that may have generational consequences.

## Graphical abstract

Changes in behavioral traits may serve as an initial response to habitat fragmentation. Utilizing aquatic fragmentation events within a single river system, we aimed to understand both whether and how behavioral traits in a population of threespine stickleback fish ( *Gasterosteus aculeatus* ) are altered when trapped in isolated pools during the breeding season compared to those in connected areas. We found that stickleback in pools differed in parental care, aggression, and risk‐taking behavior compared to stickleback from connected areas, suggesting that fragmentation events can lead to differences in phenotype expression within a breeding season and may have generational consequences.

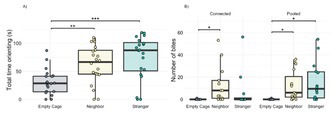

## 1 Introduction

Human‐induced change is a pervasive threat that exposes organisms to new ecological scenarios. One such scenario is habitat fragmentation, which describes a spatial discontinuity in resources or conditions necessary for occupancy and survival (Fahrig 2003; Franklin et al. 2002; Tan et al. 2023). As habitat fragmentation is expected to worsen over time (Haddad et al. 2015), there is a growing need to identify traits that can help predict whether organisms will succeed or fail within fragments. Investigating phenotypic differences, or lack thereof, in organisms found in fragmented habitats may lead to the discovery of future generalizable patterns of persistence across populations, species, and taxa.

Behavioral traits have received attention as candidates to investigate the effects of habitat fragmentation because behavior can facilitate survival during initial periods of uncertainty (Sih et al. 2011; Wong and Candolin 2015). Yet, our empirical understanding of behaviors that differ within the context of habitat fragmentation largely remains limited to movement behavior (Fardila et al. 2017). For example, a recent meta‐analysis investigating the effects of human disturbance on animal movement suggests that habitat fragmentation alone increases movement in birds and arthropods but does not change movement in mammals (Doherty et al. 2021). While understanding movement patterns can provide information on a population's ability to escape fragmentation, many individuals will be unable to remove themselves from fragmented areas, becoming increasingly susceptible to entrapment in suboptimal conditions due to resource and energy limitations. Therefore, plasticity in other behavioral traits may be necessary for survival in novel or dwindling conditions (Apfelbeck et al. 2024; Jenkins et al. 2021; Marske et al. 2023). Incorporating behaviors other than movement will improve our understanding of the selective pressures induced by fragmentation and whether populations can respond appropriately. Utilizing organisms from a single population that can be found in fragmented and non‐fragmented habitats, are unable to remove themselves from fragmented areas, and display a variety of measurable behaviors linked to survival and fitness will serve as a first step in identifying whether and how habitat fragmentation impacts behavioral phenotypes.

Parental care increases the survival and fitness of offspring, and parents should adjust their care in response to environmental conditions to maximize their own fitness and offspring success (Clutton‐Brock 2019). Therefore, understanding whether parents can alter their behavior in response to fragmentation will provide important insight into the potential for long‐term population persistence. During reproductive periods, parents engage in costly behaviors such as territory establishment, offspring defense, and care associated with offspring growth and development (incubation and food provisioning) (Clutton‐Brock 2019). These behaviors have been found to change in response to ecological stressors that may also be present in fragmented environments. For example, shifts in resource availability and interactions with predators alter investment in offspring (Hinam and Clair 2008; Lima 2009), while increased conspecific densities can increase aggression between individuals (Banks et al. 2007; Knell 2009; Yoon et al. 2012). Importantly, behavioral changes in parents can influence offspring life history, morphology, and behavioral traits (Tariel et al. 2020), suggesting that parenting behavior may also serve as a predictor of population outcomes. While parental care is widely studied across taxa, comparative work examining parental care and its effects on offspring in fragmented habitats has received less attention.

In this experiment, we examined whether habitat fragmentation is a contributing factor to behavioral phenotype expression using the Threespine stickleback fish ( *Gasterosteus aculeatus* ) as a model. Stickleback exhibit paternal‐only care during the breeding season (May–September), with a full parenting cycle ranging between 10 and 14 days (Barrett and Stein 2024; Stein and Bell 2012). During this time, males exhibit a number of behaviors associated with generational fitness, such as territory establishment, territory defense, courtship, and offspring care in the form of fanning developing embryos (Ostlund‐Nilsson et al. 2006; Wootton 1972). Following hatching and several days of paternal care, offspring disperse from the nest, form shoals with conspecifics, and compete for available resources. Stickleback breed in shallow areas with reduced flow, making adults and their offspring more susceptible to entrapment in areas that become disconnected from main water channels. Entrapment may impact stickleback behavioral evolution: after approximately 50 generations of isolation, “resident” stickleback that were disconnected from their anadromous migratory population of origin were found to be bolder and more aggressive (Ramesh et al. 2022). To date, it remains unclear whether freshwater populations of stickleback that experience natural fragmentation events display similar behavioral patterns within a breeding season.

Utilizing a river system with a history of drought in northern California, we tested the hypothesis that both adult and juvenile behavior would differ in fragmented areas. We identified stickleback in fragmented (pooled) and non‐fragmented (connected) community types and measured behaviors associated with parental care in adults and risk‐taking in juveniles. First, we recorded direct parental care behaviors linked to offspring survival. We then assessed general aggression in both territorial and parenting males by exposing individuals to different intruder contexts: a control, a known conspecific, and an unknown conspecific. During aggression assays, we recorded both the total time orienting toward each intruder type and total bites. Finally, we used both an emergence assay and a scototaxis assay to measure risk‐taking behavior in juveniles found within each community type. Here, we form our predictions under the assumptions that habitat quality and resource availability are lower in pools due to a complete lack of water flow (Bonada et al. 2020). We predicted that parenting males in pools would show a reduction in offspring care compared to stickleback in connected areas, while both territorial and parenting males would increase aggression. Additionally, we predicted that pooled juveniles would show greater risk‐taking behavior by emerging into a novel arena more often and faster than juveniles from connected areas, as well as spend more time in areas of the arena that would increase conspicuousness.

## 2 Methods

### 2.1 Site Selection

Data were collected at the south fork of the Navarro River in Philo, California (39.098173569308, −123.49980254730339; Mendocino County, CA) from May to July 2022. The Navarro River is an undammed freshwater system that relies on rainfall and snow melt during winter months to maintain water flow during summer months. In addition to natural flow rate declines, factors such as west coast drought conditions and agriculture (Hines and Kohlsmith 2012; Thompson et al. 2022) have resulted in aquatic fragmentation in the form of small, disconnected pools throughout the stickleback breeding season (May—August). These sites typically remain disconnected until reconnection in late December through January (pers. obs.).

Prior to behavioral observations and experimental manipulations, adult stickleback were located along the Navarro at sites that were categorized as either “pooled” or “connected” community types. Sites were considered pooled if stickleback were unable to enter or exit an area, while connected sites had access to flowing portions of the river. Distances between sites at the start of our study, as well as rainfall gauge data downstream from our sites (USGS Identification Number: 11468000; U.S. Geological Survey 2022), suggest that stickleback were trapped in pools throughout the breeding season. Visual observations were used to identify community composition. In all sites, stickleback and California roach ( *Lavinia symmetricus* ), but not salmonid (*Oncorhynchus* spp.) or sculpin predators (*Cottus* spp.), were directly observed. For methods described below in [Offspring Care] and [Aggression], two replicates were categorized as pooled and three were categorized as connected. For methods described in [Risk‐taking], an additional pool containing juveniles, but not adults, was added for a total of three replicates. Within each replicate, adult males that exhibited territoriality or fanning behavior were identified. Orange flagging tape was attached to surrounding vegetation above the water surface and placed ~30 cm outside of the territory for non‐invasive identification during behavioral observations. As the closest distance between nests in these neighborhoods was 90 cm, flagging tape did not overlap multiple territories.

### 2.2 Behavioral Observations

#### 2.2.1 Offspring Care

For a total of 10 min, two parenting behaviors were recorded for each adult male: time spent fanning and time spent within one body length of the nest. Following parenting observations, males (sample size connected: 9; pooled: 8) were gently removed from their nest using a dip net, and a mesh net was immediately placed over the nest to prevent depredation. Males were photographed for length measurements and placed in a 19‐L bucket with fresh river water. We then examined the nest to determine offspring developmental stage (eggs or fry). Nest depth, distance to shore, and nearest neighbor distances were recorded, as nest location can play a role in parental care behaviors (Stein and Bell 2015). Following all morphological, developmental, and nest characteristic recordings, we released and monitored each male until parenting resumed.

#### 2.2.2 Aggression

Adult male sticklebacks exhibit aggression throughout the parenting cycle to aid in territory establishment, courtship, and offspring defense. Therefore, males displaying territoriality or parental care behaviors were included in aggression assays. Assays were performed as in Stein and Bell (2015). Briefly, intruder males in nuptial coloration were captured via dip nets and placed in a wire cage (10 cm × 10 cm × 10 cm). This allows for visual and olfactory cues between individuals but does not allow for males to injure one another. At the start of the trial, caged intruders were gently placed in the center of a focal male's established territory (Video S1 available on Dryad). If a nest was present, intruder males were placed ~15 cm away from the nest to avoid disturbance.

Focal male aggression was measured across three different intruder treatments that were presented in random order: empty cage, known (neighbor), or unknown (stranger). Strangers were collected from different community types (i.e., pooled males were collected and exposed to connected males), while neighbors were collected within the same community and with territories near the focal male. Following netting, intruder males were placed in the mesh cage, lowered into a 19‐L bucket containing fresh river water, and transported to the focal male's territory. Behavioral trials began as soon as the cage was placed in the focal male's territory and ran for a total of 120 s. Measured observations included time spent orienting and the number of bites at the cage. Following assays, both focal and intruder males were photographed for length.

Due to small population sizes resulting from drought conditions at the time of measurement (sample size connected: 9, 13, 11; pooled: 11, 10, 10), focal males were also used as intruders. Males were not used as intruders until observations associated with those individuals were complete. If males had a nest at the time of collection, a mesh net was placed over the nest to avoid depredation. Intruder males were utilized for a maximum of three trials.

#### 2.2.3 Juvenile Risk‐Taking

To assess risk‐taking behavior in pooled and connected sites, juvenile stickleback were netted from each site (sample size connected: 26; pooled: 26) and immediately placed in a refuge pod. We constructed the refuge pod using a combination of opaque PVC pipe (8.9 cm × 17.8 cm) and PVC caps (7.6 cm). An exit hole (3.8 cm) was drilled into the side of the PVC pipe and blocked with a rubber stopper. Fishing line was attached to the rubber stopper so that it could be pulled from a distance. The pod was then placed in the center of a scototaxis testing arena, a 19‐L bucket (25.4 cm × 36.2 cm) with one half of the arena covered in black duct tape and the other covered in white duct tape. Once the pod was placed in the arena, each fish was given a 120‐s acclimation period. Then, the rubber stopper was pulled and individuals were allotted 180 s to exit the pod. If individuals did not exit the pod within the allotted timeframe, they were given a maximum latency to emerge time of 180 s and gently released into the arena by the observer. Following the exit or gentle release from the pod, the assay continued for an additional 180 s. At the completion of the assay, the proportion of time spent in the white portion of the arena was recorded, as time spent in white is associated with greater risk‐taking behavior (Hellmann et al. 2020; Maximino et al. 2010).

### 2.3 Statistical Analysis

Data were analyzed in R Studio Version 2023.03.0. Both data and data analysis methods are available on Dryad. Residuals were checked for normality prior to model interpretation. To assess how habitat fragmentation impacts behavioral phenotypes, we utilized both linear mixed models and generalized linear mixed models from the “lme4” package (Bates et al. 2015). Unless stated otherwise, we performed significance testing using the “lmerTest” package (Kuznetsova et al. 2017) and both conditional and marginal *R* ^2^ values were determined using the “MuMIn” package (Bartoń 2010). We performed post hoc tests using emmeans from the “emmeans” package (Lenth et al. 2018), which included a false discovery rate (FDR) test correction to account for multiple comparisons between levels. Finally, means and standard errors across treatments and levels were calculated for reporting in Section 3.

We first investigated whether fragmentation impacted direct parental care behaviors such as time spent on the nest and total time spent fanning while on the nest. It is possible that males that fanned more simply spent more time at their nest; therefore, we also calculated and analyzed the percentage of time on the nest that was spent fanning. All models included community type (pooled, connected) as a fixed effect, male length and nest depth as covariates, and site ID as a random effect.

For aggression behaviors in adult territorial and parenting males, we used a linear mixed model (time orienting) and a generalized linear mixed model with a Poisson distribution (number of bites). A Type III Wald chi‐squared test from the “car” package (Fox et al. 2001) was used for significance testing. Both models included community type (pooled, connected), intruder treatment (empty cage, neighbor, stranger), the interaction between community type and intruder treatment, and parenting stage (territorial, parenting) as fixed effects. Covariates included exposure order, focal fish length, and nest depth. Site ID and male ID served as random effects.

Finally, we examined whether risk‐taking behavior differed between juveniles found in connected and pooled community types. We used a Pearson's chi‐squared test to determine whether there were differences in the number of fish that willingly entered the scototaxis arena and the number of fish that were gently released into the arena. We then ran linear mixed models for our behaviors of interest. For latency to emerge, only individuals that willingly entered the arena were included in our analysis. Our latency to emerge model included community type as a fixed effect and site ID as a random effect. For the proportion of time spent in the white portion of the arena, we hypothesized that behavioral phenotypes may differ between those who willingly emerged and those that were gently released from the pod. Therefore, we included both community type and emergence type (emerged/nonemerged) as fixed effects. Site ID was included as a random effect.

## 3 Results

### 3.1 Offspring Care

**FIGURE 1:**
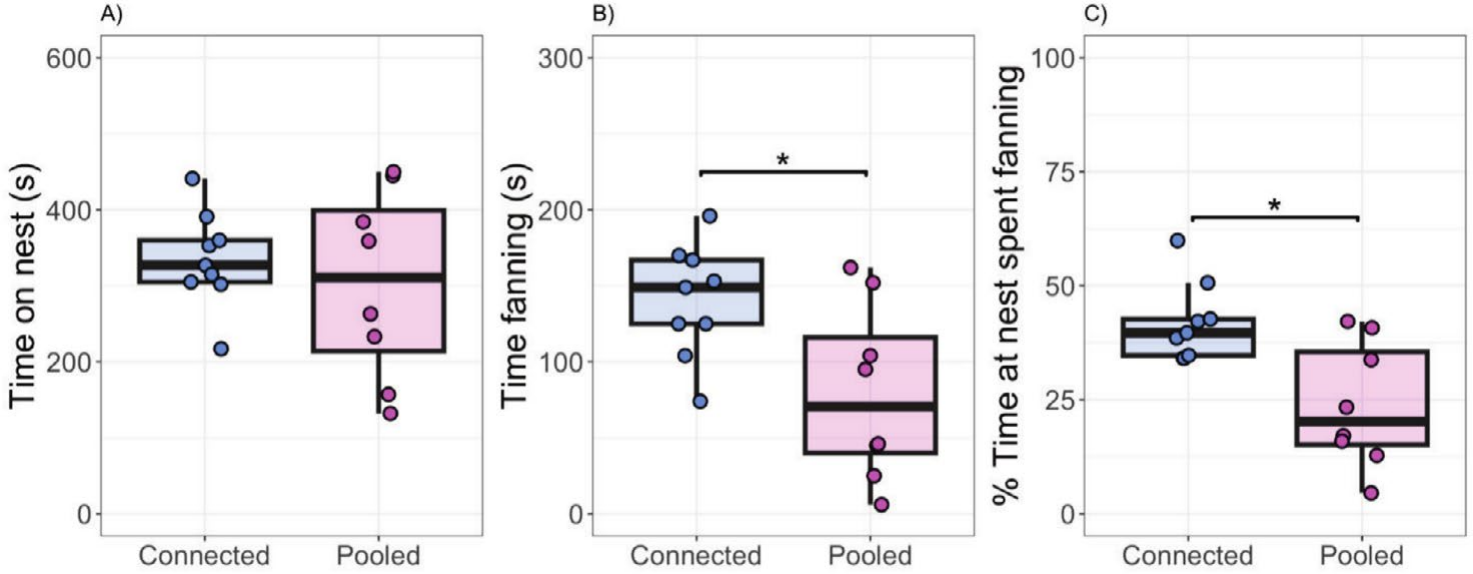
Parental care behaviors of adult male stickleback in connected and pooled communities. (A) Time spent on the nest, (B) total time fanning, and (C) percentage of time on the nest, which was spent fanning. Boxplots include medians, interquartile ranges, and whiskers representing 1.5× interquartile ranges. Asterisks represent significant effects (*p* < 0.05).

No differences were observed in total time spent at the nest (*F*_1,12_ = 0.43, *p* = 0.53) (Table 1; Figure 1A). However, total time spent fanning (*F*_1,12_ = 5.06, *p* = 0.04) and the percentage of total time on the nest which was spent fanning (*F*_1,12_ = 9.07, *p* = 0.01) differed across community types. Generally, males from pooled communities showed reduced fanning behavior compared to males from connected areas (pooled: 79.37 ± 20.52 s; connected: 140.33 ± 12.43 s) (Table 1; Figure 1B). Additionally, pooled males exhibited a reduction in the proportion of time spent fanning while at their nest compared to males found in connected areas (pooled: 23.81% ± 4.86%; connected: 41.85% ± 2.87%; Table 1; Figure 1C).

**TABLE 1:** Linear mixed models of parental care behaviors in adult male stickleback for total time on nest, total fan at nest, and percentage of time on the nest spent fanning.

Factor	Total time on nest			Total time fanning			Percentage of time on nest spent fanning		
	*F*	df	*p*	*F*	df	*p*	*F*	df	*p*
Community type (connected, pooled)	0.43	1, 12	0.53	**5.06**	**1, 12**	**0.04**	**9.07**	**1, 12**	**0.01**
Length	0.77	1, 12	0.40	0.11	1, 12	0.74	< 0.001	1, 12	0.98
Nest depth	< 0.001	1, 12	0.99	0.06	1, 12	0.79	0.66	1, 12	0.43

Random effect	LRT		*p*	LRT		*p*	LRT		*p*
Site	0		1	0		1	0		1
Marginal *R* ^2^	0.08			0.28			0.39		
Conditional *R* ^2^	0.08			0.28			0.39		

*Note:* Includes likelihood ratio test values (LRT) and both marginal and conditional *R* ^2^ values. All significant effects (*p <* 0.05) are in bold.

### 3.2 Aggression

**FIGURE 2:**
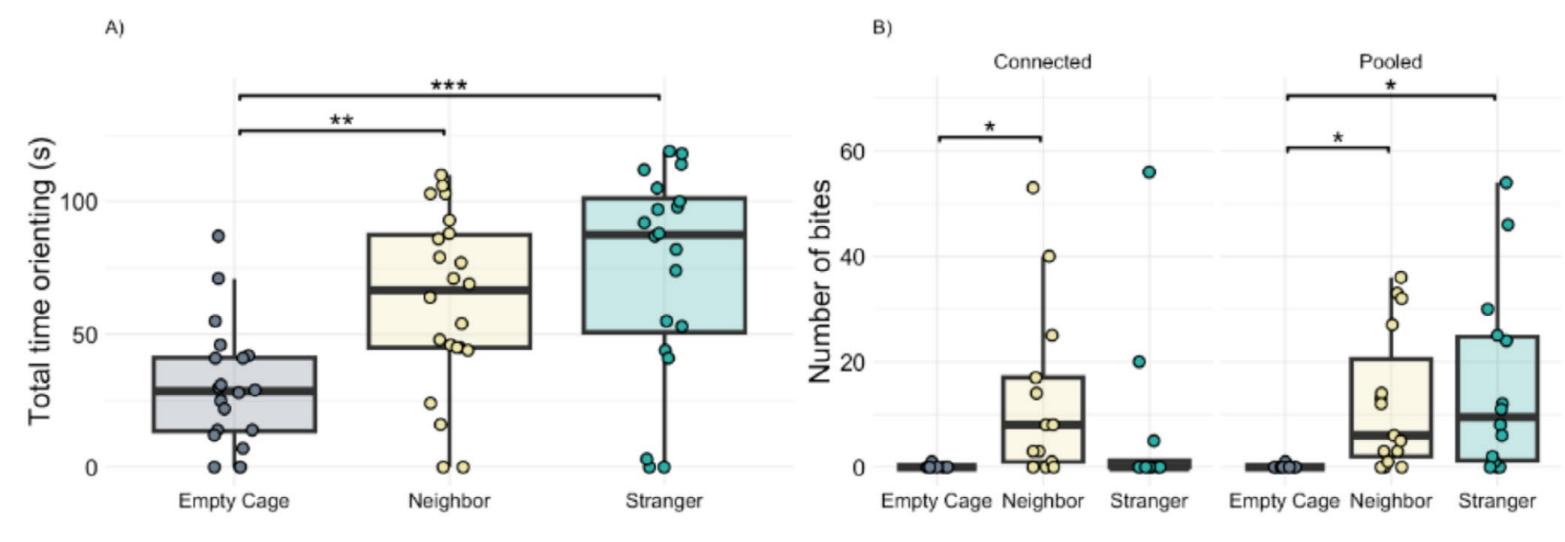
Aggression behaviors of territorial and parenting stickleback. (A) Total time orienting based on intruder type and (B) number of bites toward intruder types in connected and pooled communities. Boxplots include medians, interquartile ranges, and whiskers representing 1.5× interquartile ranges. Asterisks represent significant effects (*p* < 0.05).

Community type alone was not a contributing factor to total time spent orienting (*F*_1,2.99_ = 0.001, *p* = 0.99) (Table 2), but orienting did differ based on intruder treatment (*F*_2,33.20_ = 8.95, *p* = < 0.001) (Table 2; Figure 2A; Figure S1A available on Dryad). Generally, males spent more time orienting toward a conspecific intruder than an empty cage (Empty Cage: 29.75 ± 5.22 s, Neighbor: 62.32 ± 7.07 s, Stranger: 74.1 ± 8.78 s). There was no interaction between community type and intruder treatment in our model (*F*_2,32.77_ = 2.77, *p* = 0.15). However, visual inspection of our data suggested that individuals from one community type may be driving differences in our intruder treatment. Using a post hoc test to further examine this relationship, we found a pattern in pooled males (Figure S2 available on Dryad), who spent more time orienting toward neighbors and strangers than an empty cage alone (cage: 20.36 ± 5.15 s; neighbor: 66.2 ± 9.74 s; stranger: 84.1 ± 11.02 s), compared to males from connected areas that did not differ in time spent orienting toward any intruder (cage: 41.2 ± 8.57 s; neighbor: 59.08 ± 10.42 s; stranger: 64.10 ± 13.47 s).

**TABLE 2:** Linear mixed models of total time orienting and total bites in territorial and parenting adult male stickleback.

Factor	Total time orienting			Total bites		
	*F*	df	*p*	*χ* ^2^	df	*p*
Community type (connected, pooled)	< 0.001	1, 2.99	0.99	0.01	1	0.92
Intruder treatment (control, neighbor, stranger)	**8.95**	**2, 33.20**	**< 0.001**	**47.26**	**2**	**< 0.001**
Community type*intruder treatment	2.00	2, 32.77	0.15	**21.72**	**2**	**< 0.001**
Treatment exposure order (1, 2, 3)	0.14	2, 32.94	0.87	1.38	2	0.50
Nest stage (territorial, parenting)	0.30	1, 14.7	0.59	0.48	1	0.49
Length	0.003	1, 16.72	0.96	0.02	1	0.89

Random effects	LRT		*p*	LRT		*p*
ID	1.10		0.29	**389.55**		**< 0.001**
Site	1.45		0.23	0		1
Marginal *R* ^2^	0.28			0.78		
Conditional *R* ^2^	0.49			0.99		

*Note:* Includes likelihood ratio test values (LRT) and both marginal and conditional *R* ^2^ values. All significant effects (*p <* 0.05) are in bold.

Total bites differed based on the interaction between community type and intruder treatment (Wald chi‐squared = 21.72, *p* = < 0.001) (Table 2; Figure 2B; Figure S1B available on Dryad). Males from connected areas bit more at neighbors (13.23 ± 4.66) than they did at an empty cage (0.10 ± 0.10), while bites toward strangers (6.75 ± 4.77) did not differ from either neighbor or an empty cage. In pools, males bit significantly more at both neighbors (12.33 ± 3.41) and strangers (15.64 ± 4.73) than at an empty cage (0.067 ± 0.07).

### 3.3 Juvenile Risk‐Taking

**FIGURE 3:**
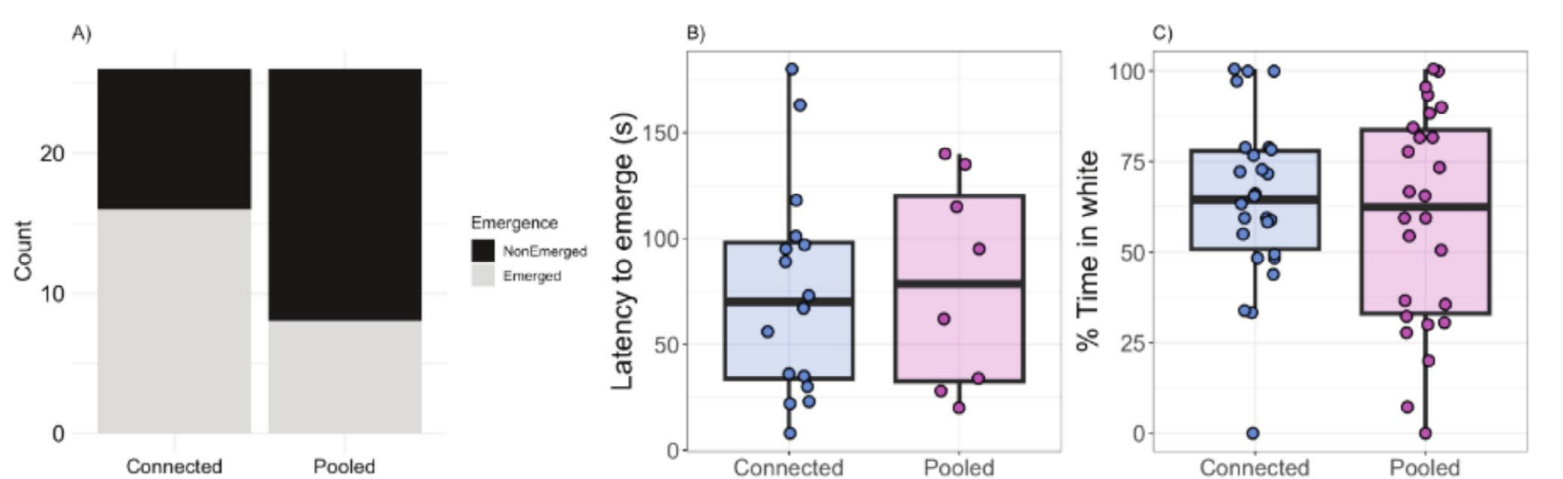
Risk‐taking behavior in juvenile stickleback. (A) Count of emerged and non‐emerged individuals based on community type, (B) latency to emerge of individuals who willingly exited the stickleback pod during the trial, and (C) proportion of time spent in the white portion of the arena. Boxplots include medians, interquartile ranges, and whiskers representing 1.5× interquartile ranges.

We observed an effect of community type on the number of juveniles that emerged within the given timeframe of 180 s, with fish from connected areas more likely to emerge into the novel arena than juveniles from pools (Pearson's chi‐squared, *p* = 0.05) (Table 3; Figure 3A). However, there were no differences in either latency to emerge (*F*_1,2.51_ = 0.14, *p* = 0.74) (Table 3; Figure 3B) or total time spent in the white portion of the arena (*F*_1,3.84_ = 0.05, *p* = 0.83) (Table 3; Figure 3C) based on the community type. Emergence was not a predictor of time spent in the white portion of the arena (*F*_1,45.5_ = 1.76, *p* = 0.19).

**TABLE 3:** Linear mixed models of latency to emerge and percent time spent in white in juvenile stickleback.

Factor	Latency to emerge			% Time in white		
	*F*	df	*p*	*F*	df	*p*
Community type (connected, pooled)	0.14	1, 2.51	0.74	0.05	1, 3.84	0.83
Emergence (Emerged/nonemerged)	—	—	—	1.76	1, 45.5	0.19

Random effect	LRT		*p*	LRT		*p*
Site	0.57		0.45	0		1
Marginal *R* ^2^	0.01			0.042		
Conditional *R* ^2^	0.2			0.042		

*Note:* Includes likelihood ratio test values (LRT) and both marginal and conditional *R* ^2^ values.

## 4 Discussion

Behavioral traits are potential predictors of population persistence in response to habitat fragmentation, yet our understanding of behavioral shifts within fragments largely remains limited to movement (Doherty et al. 2021). Parental care is tied to offspring survival (Clutton‐Brock 2019), and shifts in care may be indicative of population success or failure in response to habitat fragmentation. Here, we examined whether aquatic habitat fragmentation influences behavioral phenotypes in threespine stickleback, a fish that exhibits paternal‐only care during summer months and is likely to become trapped without opportunities for dispersal. We found that offspring care and aggression in adult males, as well as risk‐taking in juveniles, differed across community types. In pools, parenting males exhibited a reduction in fanning behavior, both territorial and parenting males were more aggressive toward unknown conspecific intruders, and juveniles were more hesitant to emerge into a novel environment. Altogether, our results suggest that fragmentation can contribute to behavioral phenotype expression, particularly in reproductive behaviors during the breeding season.

We found that time spent fanning while on the nest, a critical behavior for offspring development in stickleback, was significantly lower in males from pools than males from connected areas. Different ecological factors have been found to change fanning behavior in lab‐reared stickleback and may contribute to our observed patterns in the field. One potential factor is temperature. Currently, the relationship between temperature and its effects on fanning behavior in stickleback is unclear. Males prefer to nest under higher temperatures (Bakker and Mundwiler 2021) and both higher temperatures and higher dissolved oxygen levels have been associated with faster embryo development and increased survival rates in offspring (Bakker and Mundwiler 2021). Therefore, it is possible that males in pools experienced relaxed fanning needs due to decreased embryonic development time. However, increasing global temperatures during summer months can also challenge the maximum thermal tolerance of aquatic organisms (Johnson et al. 2024) and potentially decrease levels of dissolved oxygen needed for offspring development. As a result, parents found in areas with little to no flow, such as pools, may be experiencing greater energetic demands and are unable to compensate for shifts in dissolved oxygen levels. In a previous laboratory study with stickleback from this population, males exposed to a simulated heatwave reduced the time spent fanning at their nest compared to males with no heatwave experience after the heatwave had ended (Barrett and Stein 2024), suggesting that temperature alone could account for decreases in parental care. We detected temperature differences across community types (connected: 21°C; pooled: 24°C), but as temperature and community type are confounded and there are no direct measurements of dissolved oxygen in this study, we are unable to tease apart the independent impacts of fragmentation, temperature, and dissolved oxygen. Future work on the interplay between fragmentation, nest‐site selection, and offspring development in the context of interactions between parental care and offspring needs can provide insight into mechanisms underlying the long‐term consequences of fragmentation for populations.

Alternatively, a reduction in time fanning while at the nest, but not a reduction in total time spent at the nest, may indicate a shift in parenting strategies from direct care to vigilance. In some instances, predation rates have been found to increase within fragmented areas (Hoover 2006; Newmark and Stanley 2011; Ryall and Fahrig 2006; Yahner 1988), and parenting stickleback from this population display a reduction in fanning behavior in response to predator exposure (Stein and Bell 2012). Throughout the course of our study, we did not directly observe fish predators at our sites that could consume adult stickleback. However, this does not exclude the predation of adults stemming from avian predators or the potential predation of nests from both conspecifics and heterospecifics in an isolated and potentially resource‐limited environment. Together, our findings broadly suggest that stickleback may experience greater reproductive challenges when trapped in fragments. Future empirical studies, as well as a meta‐analysis, will provide further insight into the effects of fragmentation on parental care and the selective pressures driving changes in care.

Because aggression can aid in protecting resources and gaining information about intruders (Christensen and Radford 2018; Werba et al. 2022), we predicted that fragmentation would lead to increased aggression in stickleback. We found that total time orienting and total bites did not differ based on community type alone, which may result from similar population densities across our sites or territory size adjustments of stickleback found in pools (Stanley and Wootton 1986). However, biting behavior did differ depending on both community type and intruder treatment. Males from connected areas bit more at neighbors than an empty cage, while bites toward strangers were intermediate and did not differ from either treatment. In pools, males exhibited similar biting behavior toward both types of conspecific intruders compared to bites at an empty cage. Diverging responses toward strangers across community types may be attributed to risk (Müller and Manser 2007; Temeles 1990, 1994). In connected areas, the number of strangers present in the environment exceeds those of neighbors and strangers are less likely to be in competition for resources from a nesting male because they have access to territories, mates, and food. As a result, the physical and energetic costs associated with biting may be greater than the risk of a territory intrusion by a stranger, leading to a reduction in aggression. In contrast, males in pools may encounter fewer strangers and experience greater resource limitations because of isolation from the main portion of the river. Strangers can therefore pose a greater risk to a male during a territory intrusion, leading to increased aggression. Together, our findings highlight that habitat fragmentation contributes to subtle shifts in aggression toward conspecifics, providing a potential mechanism for large‐scale changes in social behavior. Investigating the causes and consequences of altered aggression patterns will serve as an appropriate next step in understanding the impact of habitat fragmentation on social strategies during parental care (Banks et al. 2007).

As fragmentation may lead to increased competition for resources, we predicted that juveniles in pooled areas would show increased risk‐taking behavior compared to those from connected areas. Contrary to our predictions, no differences were observed in either latency to emerge or time spent in conspicuous portions of the arena across community types. Instead, juveniles from pools were less likely to emerge into a novel arena, suggesting greater risk aversion compared to juveniles from connected sites. One reason for differences in willingness to emerge, but not other behaviors, is that emergence is a form of generalized risk aversion and conspicuousness is a form of acute risk aversion. For example, while our study did not assay individuals multiple times, generalized risk aversion is indicative of baseline behavioral syndromes, where “shy” individuals are predicted to show reduced risk‐taking behavior in new ecological conditions (Cote et al. 2010; Pamela Delarue et al. 2015; Sih et al. 2004). Therefore, it is possible that juveniles from pooled areas may be more susceptible to entrapment in the future, as they may be less likely to move away from their home sites upon reconnection to the main river body. In contrast, behavioral conspicuousness, such as time spent in white, may be considered an acute risk response because individuals need to tailor their behavior to remain competitive for resources or avoid ecological stressors. Here, our assay occurred without the presence of an ecological stressor, suggesting that acute risk responses would likely remain unchanged. Our study could not determine whether differences in juvenile behavior might be due to changes in parental care or direct juvenile experience. Studies in the lab have shown that parenting male stickleback exposed to predation risk produce offspring with fewer risk‐taking behaviors (Stein et al. 2018; Stein and Bell 2014); therefore, it is possible that both changes in parental care due to fragmentation, as demonstrated here, as well as personal experience may influence both competitive and dispersal abilities of juveniles. Further exploration of long‐term consequences of parental care on juvenile behaviors, as well as behavioral syndromes and their role in risk‐taking and movement, will help to determine whether suites of behavioral traits both within and across generations predict individual success in fragmented areas.

Aquatic organisms are especially susceptible to fragmentation due to their limited ability to escape. It is estimated that over half of the world's river systems experience declines in water flow (Messager et al. 2021), which can result in inaccessible passageways and the establishment of isolated habitat patches. Human activity has exacerbated aquatic habitat fragmentation by both prolonging flow reductions in non‐perennial systems and triggering flow reductions in historically perennial systems (Datry et al. 2023). Changes in these systems are likely to increase the frequency and duration of entrapment of aquatic organisms, yet aquatic organisms receive less attention than taxa such as birds and mammals (Fardila et al. 2017). Investigating whether aquatic fragmentation differs from terrestrial fragmentation, as well as increasing the representation of taxa across studies, will provide a holistic view of factors driving species loss across different habitats.

In conclusion, our work highlights that behavioral phenotype expression differs in an aquatic organism found in fragmented areas of a river system. A reduction in direct offspring care and changes in aggression in breeding males, coupled with risk aversion in juveniles, suggests that individuals may face greater reproductive and early‐life stage challenges in fragmented habitats. Identifying whether such behavioral change is linked to negative population outcomes or serves as a mechanism to mediate the effects of fragmentation (Donelan et al. 2020; Duckworth 2009) will serve as an area of future research. Our results provide a foundation to elucidate selective pressures acting on parenting phenotypes within aquatic fragments, as well as determine whether parental effects negatively or positively impact subsequent generations.

## Author Contributions

**F. Leri:** conceptualization (lead), data curation (lead), formal analysis (equal), funding acquisition (supporting), investigation (lead), methodology (lead), project administration (lead), resources (supporting), software (equal), supervision (supporting), validation (equal), visualization (equal), writing – original draft (lead), writing – review and editing (equal). **L. R. Stein:** conceptualization (supporting), data curation (supporting), formal analysis (equal), funding acquisition (lead), investigation (supporting), methodology (supporting), project administration (lead), resources (supporting), software (equal), supervision (lead), validation (equal), visualization (equal), writing – original draft (supporting), writing – review and editing (equal).

## Conflicts of Interest

The authors declare no conflicts of interest.

## Supporting information


**Figure S1:** (A) Total time orienting at an intruder and (B) total bites when exposed to an empty cage, neighbor, and stranger.
**Figure S2:** Total time spent orienting at an intruder across connected and pooled community types.

**Data S1:** ece372099‐sup‐0002‐Supinfo2.csv.

**Data S2:** ece372099‐sup‐0003‐Supinfo3.csv.

**Data S3:** ece372099‐sup‐0004‐Supinfo4.csv.

**Video S1:** ece372099‐sup‐0005‐VideoS1.mp4. Video of aggression assay in adult threespine stickleback.

## Data Availability

Both data and data analysis methods are available on Dryad and can be accessed with the provided link https://doi.org/10.5061/dryad.n8pk0p35h.
