# Supplementary material for: Feeling Disconnected: River Fragmentation Alters Parenting, Aggression, and Risk‐Taking in Threespine Stickleback
Source: Ecol Evol. 2025 Sep 15;15(9):e72099. doi: 10.1002/ece3.72099 (PMC12434314; doi:10.1002/ece3.72099)
Supplement: Supplementary file 1 — Figure S1: (A) Total time orienting at an intruder and (B) total bites when exposed to an empty cage, neighbor, and stranger. Figure S2: Total time spent orienting at an intruder across connected and pooled community types. [file ECE3-15-e72099-s003.docx]

APPENDIX


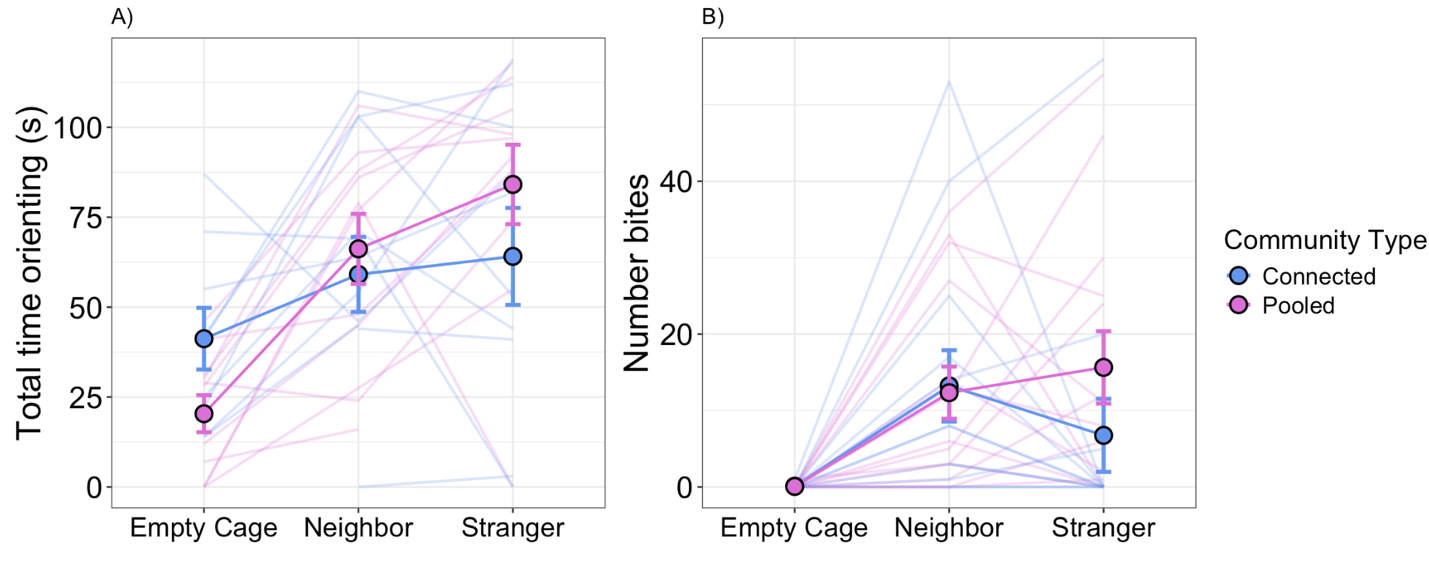


**Figure A1:** A) Total time orienting at an intruder and B) Total bites when exposed to an empty cage, neighbor, and stranger. Blue points represent group means with standard errors for males in connected sites, while pink points represent males from pooled sites. Lines represent individuals within groups.


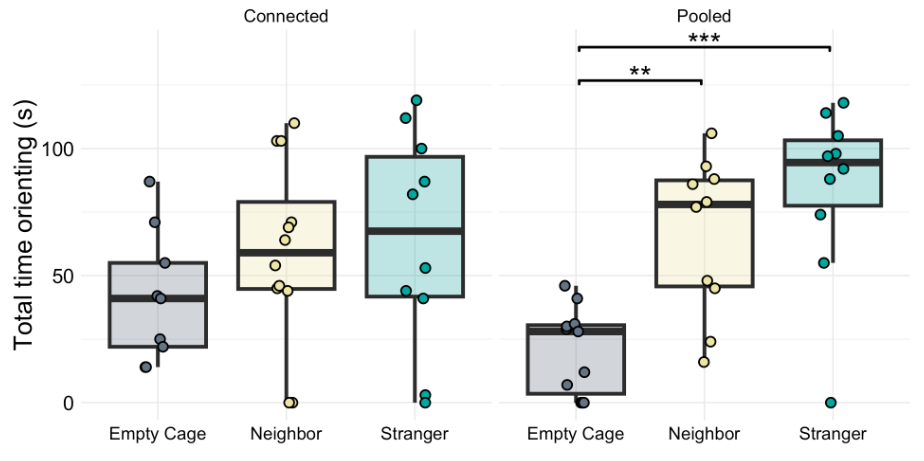


**Figure A2:** Total time spent orienting at an intruder across connected and pooled community types.
